# The Healing of Rodent Cancer by Electricity

**Published:** 1894-09

**Authors:** 


*The Healing of Rodent Cancer by Electricity.* By J. INGLIS PARSONS, M.D. Pp. 82. London: JOHN BALE & SONS. 1893.

We heartily welcome this small and well-written monograph by Dr. Inglis Parsons on a subject with which his name has been associated for some years. Any remedy for malignant new growths deserves careful investigation, for our therapeutic resources in this direction are practically *nil;* but so many vaunted cures for cancer have been brought before the notice of the profession that many of us have been inclined to prejudge Dr. Parsons's method as another chimera. This practical account of his treatment is full of interest, for sufficient time has elapsed for the author to review his results and to invite the criticism of his professional brethren with every confidence that his claims are well founded. After a succinct account of the pathology and morbid anatomy of rodent ulcer, he passes on to the very practical question of batteries, and his brief notes on these and how to use them are clear and to the point.

After describing the technique of the operation, he reports four cases, and gives illustrations from photographs of a patient before and after treatment, which leave little room for doubt that he is doing good work.

